# PASTA kinase signaling regulates peptidoglycan synthesis in *Enterococcus faecalis* by direct inhibition of UDP-N-acetylglucosamine 1-carboxyvinyl transferase activity

**DOI:** 10.1128/mbio.00593-25

**Published:** 2025-04-24

**Authors:** Carly A. Mascari, Dušanka Djorić, Christopher J. Kristich

**Affiliations:** 1 Department of Microbiology and Immunology, Center for Infectious Disease Research, Medical College of Wisconsin 5506, Milwaukee, Wisconsin, USA; Universite de Geneve, Geneva, Switzerland

**Keywords:** *Firmicutes*, *Enterococcus faecalis*, peptidoglycan synthesis, PASTA kinase

## Abstract

Proper control of bacterial peptidoglycan (PG) synthesis is critical to balance growth, cell division, and stress responses with other energetic needs of the cell. The first committed step of the PG biosynthetic pathway is catalyzed by UDP-N-acetylglucosamine 1-carboxyvinyl transferases (UNAG-CTases). The genomes of most *Firmicutes* encode two UNAG-CTase homologs: MurAA (or MurA/MurA1) and MurAB (or MurZ/MurA2). The primary UNAG-CTase in many *Firmicutes* (MurAA) is regulated by proteolysis in response to signals sensed by transmembrane kinases containing PASTA domains through the action of the kinase substrate IreB, impacting the amount and/or rate of PG synthesis. However, the secondary UNAG-CTases in *Firmicutes* do not appear to be controlled by proteolysis, and their regulation remains unknown. We sought to determine if signaling via IreK, the PASTA kinase in the opportunistic pathogen *Enterococcus faecalis*, might also regulate PG synthesis by the secondary UNAG-CTase (MurAB). Using genetic and biochemical approaches, we found that IreK-mediated phosphorylation of IreB was essential in the absence of MurAA, confirming that IreB regulates additional targets beyond MurAA. We demonstrated that the secondary UNAG-CTase, MurAB, is one such target and that IreB directly regulates the catalytic activity of MurAB via phosphorylation-modulated direct physical interaction to impact PG synthesis in *E. faecalis*. Hence, our work establishes not only a new regulatory target for the IreK-IreB signaling axis and a new mechanism of action for IreB but also the first described regulatory mechanism for a MurAB homolog in any organism, a mechanism that is distinct from the established paradigm for the primary UNAG-CTases.

**IMPORTANCE:**

Peptidoglycan (PG) is a critical mesh-like polymer that provides osmotic support and structure to the bacterial cell wall, and regulation of its synthesis is essential for proper cell growth, division, and stress responses. In *Firmicutes*, control of PG synthesis is known to occur through the regulation of the primary UNAG-CTase by proteolysis in response to signals mediated by the transmembrane PASTA kinase. *Firmicutes* also encode a secondary UNAG-CTase homolog whose regulation has remained unknown. Our results demonstrate a new mechanism for the regulation of PG synthesis in *Firmicutes*—direct inhibition of the enzymatic activity of the secondary UNAG-CTase by the PASTA kinase-IreB signaling axis via phosphorylation-modulated direct physical interaction between IreB and the secondary UNAG-CTase in *Enterococcus faecalis*.

## INTRODUCTION

Peptidoglycan (PG) is a critical mesh-like polymer, comprising chains of repeating N-acetylglucosamine and N-acetylmuramic acid disaccharides cross-linked via short peptides, that provides osmotic support and structure to the bacterial cell . Synthesis and remodeling of this polymer are essential for cell growth, division, and stress response—but PG synthesis must be balanced with other cellular processes for which resources are shared (1). For example, the UDP-N-acetylglucosamine (UDP-GlcNAc) required for PG synthesis is derived from fructose-6-phosphate, an important intermediate of central carbon metabolism. UDP-GlcNAc, itself, is also used for the synthesis and modification of cell surface glycopolymers. Utilization of UDP-GlcNAc in the first committed step of the PG synthesis pathway is an important regulatory point in many organisms. For example, in *Mycobacterium tuberculosis,* the activity of MurA, the UDP-N-acetylglucosamine 1-carboxyvinyl transferase (UNAG-CTase) that catalyzes this reaction, is activated by a regulatory protein called CwlM in response to nutrient availability (2). During starvation, MurA is not activated by CwlM, and as a result, peptidoglycan synthesis is restricted. Additionally, the *Escherichia coli* homolog of MurA has been demonstrated *in vitro* to be inhibited by UDP-MurNAc, the product of a subsequent reaction in the PG synthesis pathway (3). Crystallographic studies further suggest that *Enterobacter cloacae* MurA binds UDP-MurNAc to form a reversible dormant complex (4). Together, these studies informed a model in which MurA is feedback inhibited in these two gram-negative bacteria in response to the cellular ratio of UDP-MurNAc:UDP-GlcNAc such that MurA activity (and therefore allocation of UDP-GlcNAc for PG synthesis) is restricted when UDP-MurNAc levels are sufficiently high (3, 4).

Low-GC content gram-positive bacteria encode two MurA homologs that catalyze the first committed step of PG synthesis, MurAA (also known as MurA or MurA1) and MurAB (also known as MurZ or MurA2) (5–7). MurAA, which is more similar by amino acid identity to the single-copy MurA enzyme encoded in most proteobacteria (5), tends to be considered the “primary” homolog in most organisms studied, in that mutants lacking the primary homolog exhibit growth defects (*Staphylococcus aureus* [8] and *Enterococcus faecalis* [9]) or cannot grow at all (*Bacillus subtilis* [10], *Bacillus anthracis* [11], and *Listeria monocytogenes* [12]), whereas MurAB homologs are often dispensable in standard laboratory conditions.

Recently, the cellular abundance of MurAA in several low-GC gram-positive organisms (including *L. monocytogenes* [13, 14]*, S. aureus* [15]*, B. subtilis* [14, 16], and *E. faecalis* [17]) has been demonstrated to be modulated in response to cell envelope stress via regulated proteolysis. Modulation of MurAA levels has consequences on substrate flux through the PG synthesis pathway, PG thickness, resistance to cell wall-active antibiotics, growth, and virulence (7, 13–18). Unlike MurAA, the MurAB homologs in these organisms do not appear to be regulated by proteolysis, although, intriguingly, loss of MurAB seems to result in protection of MurAA from proteolysis through an unknown mechanism (14, 17).

Regulation of MurAA proteolysis in low-GC gram-positive organisms is achieved via signaling through a transmembrane PASTA-domain-containing serine/threonine kinase that conveys extracellular stress signals to modulate phosphorylation of intracellular substrates, thereby facilitating an adaptive biological response (13–17). In the opportunistic pathogen, *Enterococcus faecalis*, this kinase is IreK (17, 19). *E. faecalis* is a commensal of the gastrointestinal tract but also a common etiological agent of many healthcare-acquired infections, including urinary tract and surgical site infections as well as endocarditis (20–24). Such infections can be difficult to treat, in part due to the intrinsic resistance of enterococci to many antibiotics and the tendency to rapidly acquire resistance to additional antimicrobials (25). The PASTA kinase IreK plays a central role in that it is required for resistance of *E. faecalis* to cell wall-active agents, such as cephalosporin antibiotics, bile salts, and lysozyme, and for long-term colonization of the mammalian gastrointestinal tract (19, 26, 27).

IreK is activated at low levels during unstressed exponential-phase growth, but cell wall stress (such as treatment with cell wall-active antibiotics [28]) triggers increased autophosphorylation and activation of the kinase. In contrast, IreK is nearly completely unphosphorylated during stationary phase (28). When autophosphorylated, IreK is activated to robustly phosphorylate downstream substrates. The full contingent of IreK substrates has not yet been fully defined, though phospho-proteomic analysis has revealed dozens of proteins become phosphorylated in an IreK-dependent manner during cell wall stress (29), suggesting potentially far-reaching effects of IreK activation. Currently, two proteins have been validated to be directly phosphorylated by IreK: a cytoplasmic protein called GpsB that plays a role in modulating IreK activation (30, 31) and the regulatory protein, IreB (28, 32, 33) that controls MurAA proteolysis (17). Unphosphorylated IreB binds MurAA and promotes its degradation via the ClpCP proteolytic complex, thereby restricting steady-state levels of MurAA (17). When IreB becomes phosphorylated by IreK, such as during exposure to cell wall-active antibiotics and to a lesser extent during active growth (28), phospho-IreB is unable to bind MurAA, resulting in MurAA accumulation that drives increased peptidoglycan synthesis.

Regulation of MurAA proteolysis is the only defined molecular function for IreB and its homologs in other organisms (13–15, 17, 18). However, recent work revealed that while IreK and IreB homologs are conserved in *Streptococcus pneumoniae*, neither of the pneumococcal MurA homologs (MurA or MurZ) is regulated by proteolysis (7). Instead, the pneumococcal PASTA kinase appears to regulate MurA/MurZ activity via some other as-yet-unknown mechanism, suggesting that IreB homologs may be capable of other regulatory functions beyond promoting ClpCP-mediated proteolysis.

To explore if IreB could regulate other targets via a mechanism distinct from ClpCP-mediated proteolysis, we investigated the role of IreB in *E. faecalis* cells lacking MurAA. We found that IreB acts to directly inhibit the enzymatic activity of MurAB, confirming that IreB not only has at least one other regulatory target but also that it can act via two distinct regulatory mechanisms. These findings also shed new light on our previous observations that MurAB and IreB are capable of physically interacting in a manner dependent on the phosphorylation state of IreB (17), the consequences of which were previously unknown. Collectively, our findings are consistent with a model in which IreK provides coordinated regulation of initiation of peptidoglycan synthesis via both MurAA and MurAB, but through distinct mechanisms, both modulated via phosphorylation of IreB and that this regulation is essential for maintenance of proper cell wall integrity and competitive fitness.

## RESULTS

### IreK-dependent phosphorylation of IreB is required for growth even in the absence of MurAA

To test the impact of IreK and IreB signaling beyond regulation of MurAA proteolysis, we attempted to generate a Δ*murAA* Δ*ireK* double-deletion strain. While neither gene is individually essential for growth, we could not construct the double mutant lacking both genes unless we simultaneously supplied MurAA from an inducible expression plasmid. The Δ*murAA* Δ*ireK* mutant only grew in the presence of the inducer, indicating that *murAA* and *ireK* are a synthetically lethal pair (Fig. S1). In contrast to what has been described in *L. monocytogenes* (13), this observation suggests that regulation of MurAA protein levels is not the only critical role of IreK signaling for growth in *E. faecalis*.

**Fig 1:**
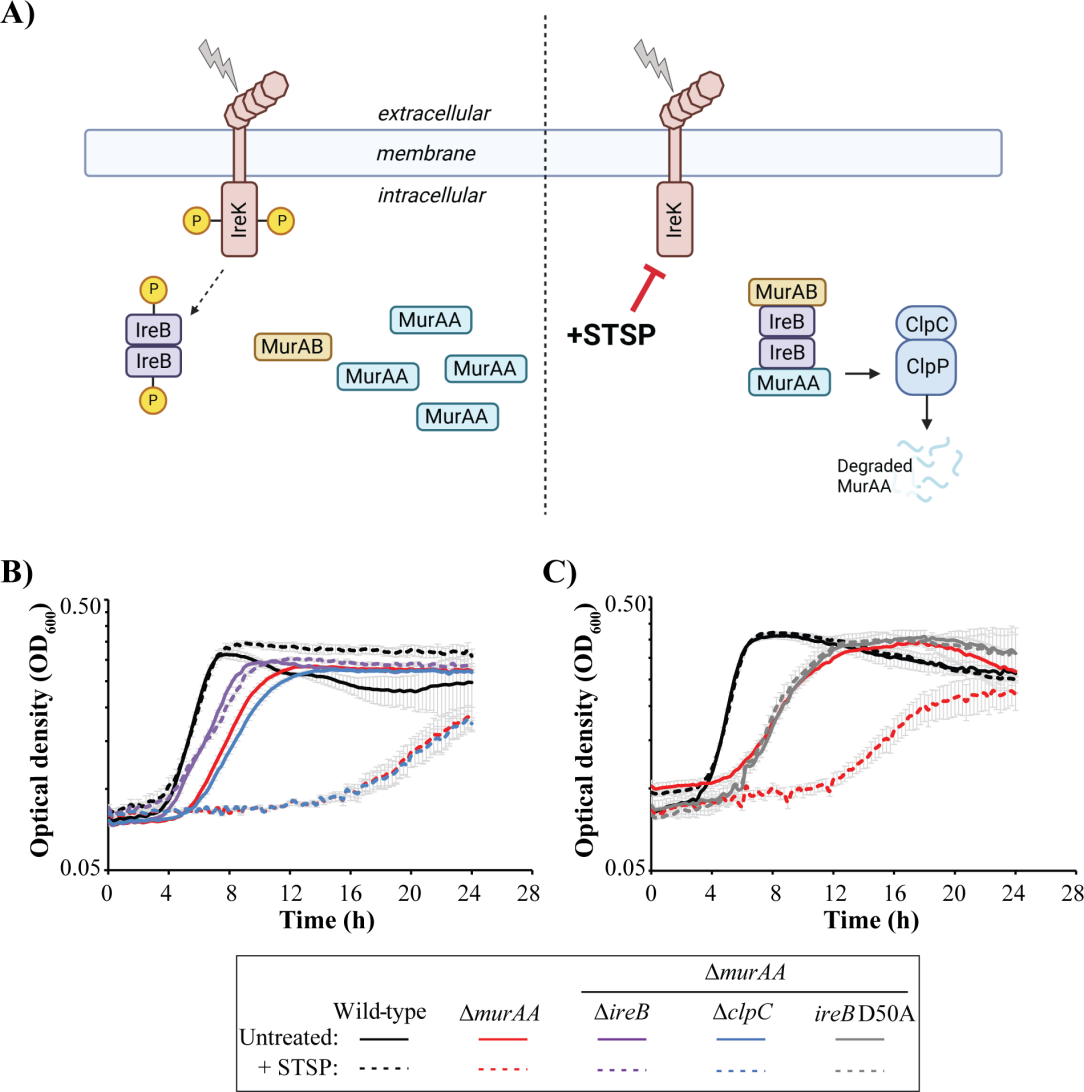
Deletion of *ireB* restores growth of the Δ*murAA* strain after inhibition of IreK with staurosporine. (**A**) Staurosporine inhibits IreK such that IreB remains unphosphorylated. Unphosphorylated IreB interacts with MurAA and facilitates ClpCP-mediated degradation of MurAA. Model created with BioRender.com. (**B** and **C**) Growth curves for the indicated *E. faecalis* strains grown at 37°C with 10 µM STSP or equivalent volume dimethyl sulfoxide (DMSO) (untreated) as a vehicle control. Panels **B** and **C** are from independent experiments, where differences between panels reflect normal variation. Growth curves represent an average of three independent replicates within each experiment, and error bars show standard deviation. Strains were wild type, OG1; ∆*murAA*, JL626; Δ*murAA* ∆*ireB*, CM32; Δ*murAA* ∆*clpC,* CM62; and Δ*murAA ireB* D50A, CM71.

To circumvent the synthetic lethality of *murAA* and *ireK,* we utilized staurosporine (STSP) (Fig. 1A). STSP is an inhibitor of eukaryotic-type serine/threonine kinases that we previously demonstrated inhibits IreK kinase activity *in vitro* (32). Note that IreK is the only eukaryotic-type kinase encoded in the *E. faecalis* genome, and the effects of STSP are therefore expected to be limited to inhibition of IreK. Consistent with this, treatment of the Δ*ireK* mutant with STSP did not result in any differences in growth (Fig. S2). The addition of STSP also did not affect growth of the wild-type strain but resulted in severely impaired growth of the Δ*murAA* mutant (Fig. 1B and C), consistent with IreK activity being critical in the absence of MurAA. While we did observe some growth of the Δ*murAA* strain in STSP-supplemented media at late time points, it is likely this is due to incomplete inactivation of IreK or potential breakdown of STSP after extended incubation. Complementation of the Δ*murAA* strain with a plasmid expressing MurAA from a constitutively active promoter restored growth in the presence of STSP (Fig. S3), confirming that the loss of MurAA was indeed driving this phenotype in the Δ*murAA* strain.

To test if the requirement for IreK activity in the absence of MurAA required signaling events specifically downstream of IreB, we challenged a Δ*murAA* Δ*ireB* double-deletion strain with STSP. The Δ*murAA* Δ*ireB* strain was not inhibited by STSP (Fig. 1B), indicating that the inability of the cells to “turn off” IreB activity via IreK-mediated phosphorylation is specifically responsible for the growth defect of the Δ*murAA* strain during STSP treatment (as opposed to any other potential IreK substrates). Because MurAA is absent from these cells, this result indicates that IreB has another role beyond modulating MurAA levels and suggests that, through this role, IreB restricts a target protein that is essential in the absence of MurAA.

In *B. subtilis,* the abundance of other ClpCP substrates was impacted by deletion of the IreB homolog ReoM (14). One such substrate was GlmS, the enzyme that catalyzes the first of four enzymatic steps of UDP-GlcNAc synthesis, thereby promoting cell wall synthesis. GlmS abundance was increased upon deletion of the IreB homolog in *B. subtilis*. However, immunoblotting analysis revealed that IreB does not promote proteolysis of GlmS in *E. faecalis* (Fig. S4).

To test more broadly whether IreB-dependent regulation of another ClpCP substrate was responsible for the phenotypes observed in Fig. 1, we generated a Δ*murAA* Δ*clpC* double-deletion strain. We reasoned that the loss of either *ireB* or *clpC* should restore growth in the Δ*murAA* background during STSP treatment if another ClpCP substrate was responsible. However, we found that unlike the Δ*murAA* Δ*ireB* strain, the Δ*murAA* Δ*clpC* strain was unable to grow in the presence of STSP (Fig. 1B). Thus, the requirement for IreB inactivation in the Δ*murAA* strain is not related to stabilization of another ClpCP substrate, suggesting that IreB possesses a distinct function. We have not ruled out the possibility that IreB could modulate proteolytic degradation for substrates of other Clp complexes, such as ClpXP (although no such function for IreB has been described), but our results described below indicate that the function of IreB goes beyond regulation of proteolysis.

### IreB inhibits the activity of MurAB *in vitro* in a phosphorylation-dependent manner

In previous work, we found that phosphorylation of IreB by IreK not only disrupted the physical interaction between IreB and MurAA (thereby preventing proteolysis of MurAA) but also disrupted the interaction between IreB and MurAB (17). MurAB is essential for growth in the absence of MurAA (18). Our findings in Fig. 1 indicated that IreB has an additional MurAA-independent function, suggesting that theIreB-MurAB interaction may impact MurAB function. To test if MurAB activity is restricted by IreB in *E. faecalis*, we introduced a plasmid expressing MurAB from a constitutive promoter into the Δ*murAA* strain and found that it restored growth in the presence of STSP (Fig. S3). Thus, overexpression of MurAB such that it can no longer be inhibited by the available pool of IreB is sufficient to overcome STSP-mediated inhibition of IreK, consistent with the model that unphosphorylated IreB inhibits MurAB to prevent growth of the Δ*murAA* strain.

**Fig 2:**
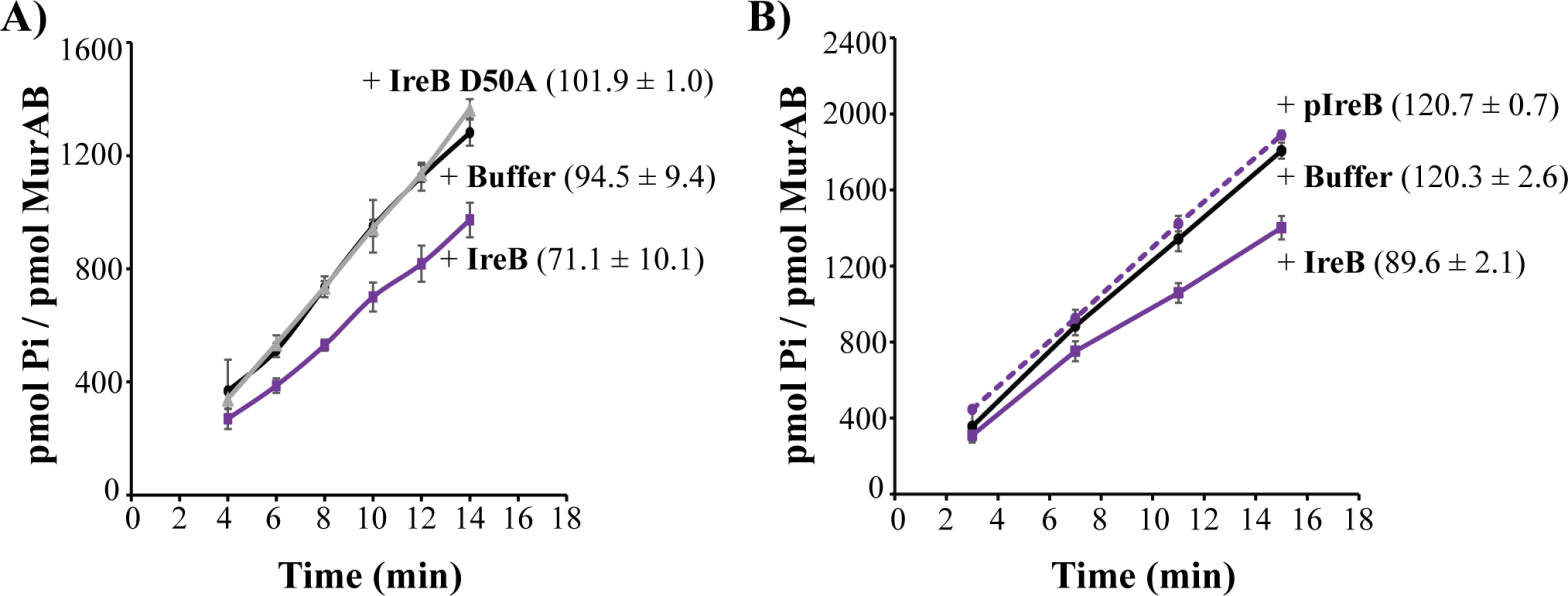
IreB inhibits enzymatic activity of MurAB in a phosphorylation-dependent manner. (**A**) UNAG-CTase activity of purified, recombinant MurAB was measured in the presence of 10-fold molar excess purified wild-type IreB (purple squares) or IreB D50A (gray triangles) or equivalent volume buffer (black circles). Data represent the average of six replicate reactions ± standard deviation. The reaction rate was calculated as the average slope for data points between 4 and 14 min and is reported in parentheses. (**B**) MurAB activity was measured in the presence of ≤20-fold molar excess of phosphorylated IreB (purple, dashed), unphosphorylated IreB (purple, solid), or buffer (black). Data represent the average of three replicates ± standard deviation. The reaction rate was calculated as the average slope for data points between 3 and 15 min and is reported in parentheses. For both panels **A** and **B**, MurAB activity was measured as the release of inorganic phosphate in the presence of saturating concentrations of UDP-GlcNAc and phosphoenolpyruvate. In some cases, error bars are present but too small to be seen.

Because MurAB cellular abundance is not impacted by IreB (17), we hypothesized that IreB instead directly regulates the enzymatic activity of MurAB. To test this, we evaluated the activity of purified, recombinant MurAB *in vitro* in the presence or absence of IreB using an enzymatic assay described previously for MurAA (9, 17). The addition of IreB led to an ~25% decrease in the rate of the MurAB-catalyzed reaction (Fig. 2A). In contrast, IreB had no effect on MurAA enzymatic activity when tested under the same conditions (Fig. S5A), indicating specificity for MurAB.

Because phosphorylation of IreB prevents the physical interaction between MurAB and IreB (17), we tested how IreB phosphorylation impacted inhibition of MurAB. After phosphorylating IreB *in vitro* using purified IreK kinase domain (as described previously [17, 30]) and removing residual ATP from these reactions, we compared the impact of phosphorylated vs non-phosphorylated IreB on MurAB activity and found that MurAB was not inhibited by the phosphorylated form of IreB (Fig. 2B). Because we did not remove (the small amount of) IreK kinase domain from our phosphorylated IreB sample prior to testing MurAB activity, as a control, we tested the impact of IreK kinase domain alone on MurAB activity and observed no effect (Fig. S5B). Together, our results indicate that IreB can regulate the enzymatic activity of MurAB in a manner controlled by IreK-dependent phosphorylation of IreB.

### An IreB D50A substitution blocks the interaction between MurAB and IreB and prevents regulation of MurAB activity

**Fig 3:**
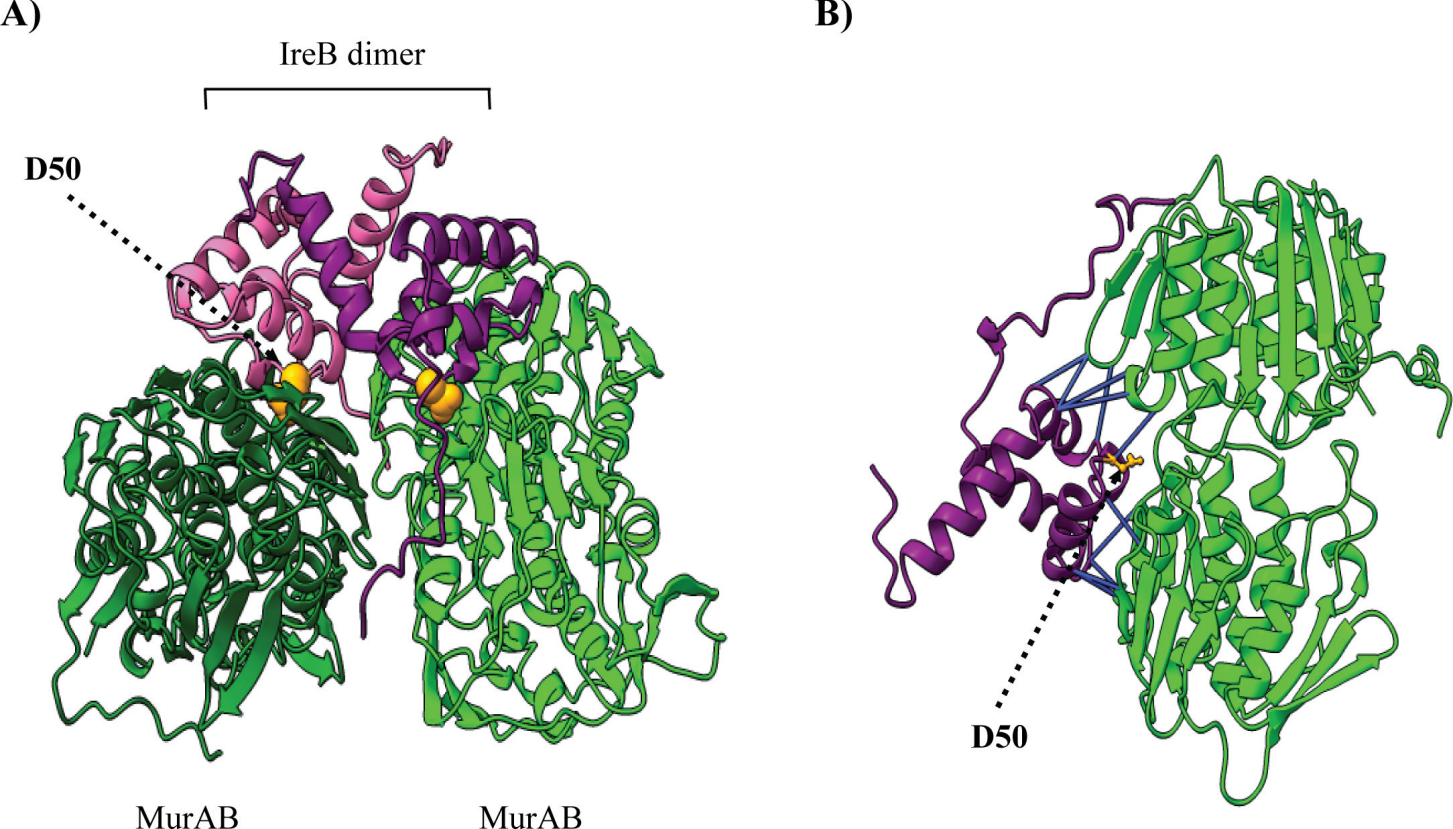
IreB D50 resides within a predicted IreB-MurAB interaction interface. (**A**) AlphaFold3 was used to model a complex between two monomers of MurAB (dark green and light green; WP_002357969.1) and an IreB homodimer (purple and pink; WP_002357937.1). D50 of each IreB monomer is indicated with orange spheres. (**B**) Only one subunit of IreB and MurAB is shown for clarity. Predicted pseudobonds between MurAB and IreB with a length <3 Å and predicted align error of ≤5 are indicated with solid blue lines. IreB D50 is labeled in orange ball-and-stick format.

IreB forms dimers both *in vitro* and *in vivo*—a feature required for its function and stability (34). In the genetic selection that originally identified IreB as a downstream substrate of IreK, we obtained IreB D50A as a loss-of-function mutant (32). IreB D50 is distal to the IreB dimer interface and located on a conserved, solvent-exposed loop of IreB that has been hypothesized to be a protein-protein interaction surface (32, 34). Modeling of the MurAB-IreB complex as a heterotetramer (IreB dimer with two MurAB monomers) using AlphaFold3 (35) predicted a high-confidence interaction complex between IreB and MurAB (Fig. 3) with a predicted template modeling (pTM) score of 0.86 (pTM >0.5 indicates overall fold is likely similar to the true structure) and an interface predicted template modeling (ipTM) score of 0.85 (ipTM >0.8 indicates a confident, high-quality prediction) (35). In the model, D50 from each IreB monomer was located at the interaction interface with a monomer of MurAB (Fig. 3A) and was predicted to be able to form contacts at <3 Å with MurAB (Fig. 3B), suggesting that IreB D50 could be important for the MurAB-IreB interaction.

**Fig 4:**
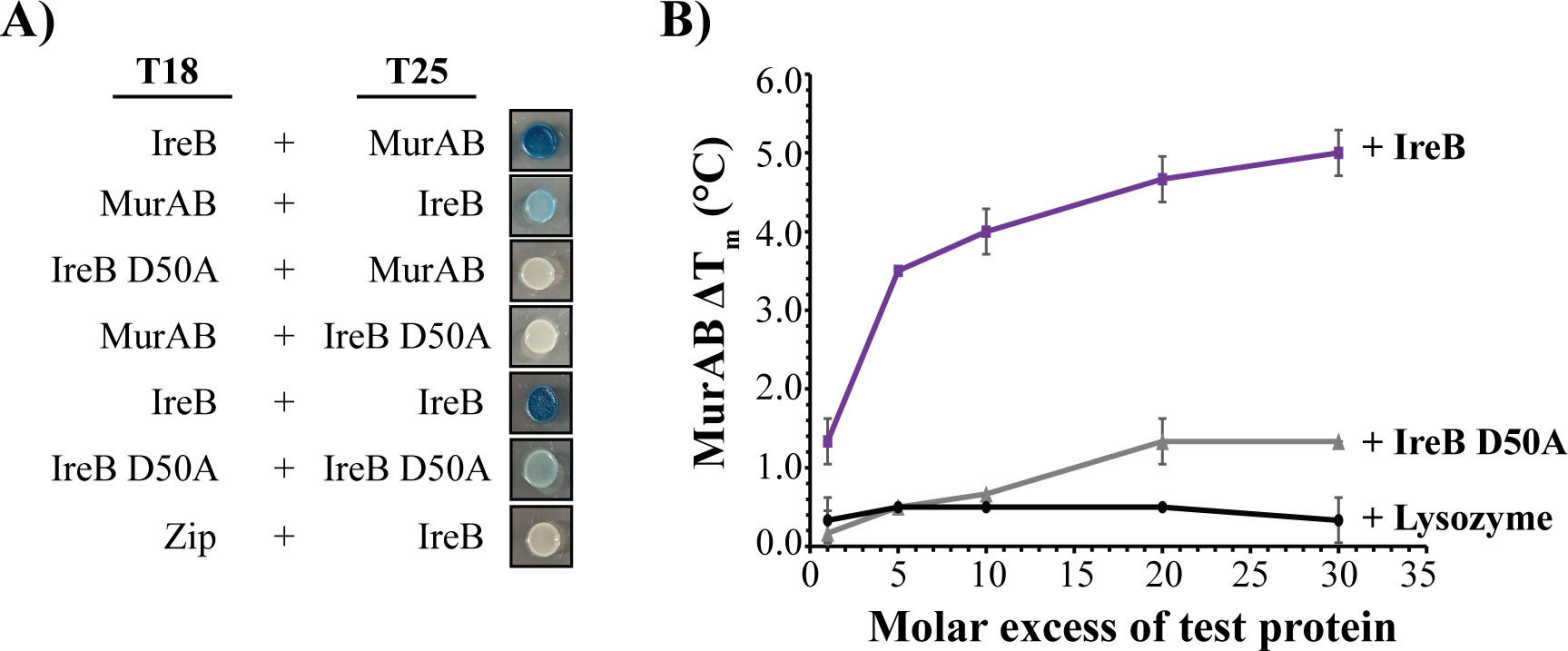
The IreB D50A mutation impairs the interaction between MurAB and IreB. (**A**) Bacterial two-hybrid assay in *E. coli* DHM1 cells co-transformed with plasmids expressing the indicated proteins fused to either the T18 or T25 domains of *B. pertussis* adenylate cyclase and spotted on media containing X-gal. Constructs containing the leucine zipper domains of GCN4 fused to the T18 and T25 domains (Zip) were used as negative controls. Images are representative of two to three replicates generated from independent transformants. (**B**) Thermal shift analysis of MurAB upon addition of wild-type IreB (purple squares), IreB D50A mutant (gray triangles), or lysozyme (black circles) as a negative, non-binding control. The change in the MurAB melt temperature (Δ*T*_*m*_) was measured using SYPRO Orange fluorescence and plotted as a function of the molar ratio of the indicated test protein relative to MurAB. Data represent the average of three independent replicates ± standard deviation.

To test this hypothesis, we used bacterial two-hybrid assays (17, 36) and thermal shift assays (TSAs) (17, 37, 38) to probe the interaction between MurAB and IreB D50A. In bacterial two-hybrid assays, MurAB and wild-type IreB exhibited interactions with each other when fused to either the T18 or T25 fragments of *Bordetella pertussis* adenylate cyclase (Fig. 4A), and in both cases, the interaction was lost upon introduction of the D50A substitution in IreB. As a control, we were able to detect self-association of the IreB D50A mutant using this approach, indicating that the loss of interaction with MurAB is not due to a failure of the IreB D50A fusions to be expressed.

As an orthogonal approach, we performed TSAs using purified, recombinant proteins (17, 37, 38), which had previously demonstrated that IreB induced thermal shift (Δ*T_m_*) for MurAB (17). We measured the change in the MurAB *T_m_* as a function of increasing molar ratios of IreB, IreB D50A, or lysozyme (non-interacting control protein) relative to MurAB (Fig. 4B). As expected, the addition of lysozyme did not result in a shift in the MurAB *T_m_*. Wild-type IreB yielded a detectable shift in the MurAB *T_m_* at equimolar IreB, with a maximum shift of 5°C. In contrast, addition of the IreB D50A mutant did not produce a detectable IreB-dependent thermal shift for MurAB until IreB D50A exceeded 10-fold molar excess, and the maximum shift detected was only ~1.5°C. Thus, while the IreB D50A mutant appears to still be capable of interacting directly with MurAB to some extent *in vitro*, the interaction is severely impaired relative to that of the interaction between wild-type IreB and MurAB.

To test if IreB D50A could influence MurAB catalytic activity, we performed enzymatic assays. In contrast to wild-type IreB, the IreB D50A mutant did not impair MurAB catalytic activity (Fig. 2A), supporting the model that the inhibition of MurAB activity observed in the presence of wild-type unphosphorylated IreB depends on a direct physical interaction between MurAB and IreB.

### Regulation of MurAB by IreB has consequences on growth and peptidoglycan synthesis *in vivo*

After validating that the IreB D50A substitution both blocks the physical interaction between IreB and MurAB and impairs IreB-mediated inhibition of MurAB *in vitro*, we asked what the phenotypic consequences of this substitution were in *E. faecalis* by introducing the D50A mutation into the chromosomal copy of *ireB*. Importantly, the steady-state abundance of IreB D50A was not reduced (Fig. S6), indicating that phenotypic differences observed with this strain are the result of functional changes in IreB. In fact, IreB levels appeared to be slightly increased in the *ireB* D50A strain, for reasons that remain unknown.

To specifically examine the impact of the IreB D50A mutation on MurAB function *in vivo,* we introduced the *ireB* D50A substitution into the native *ireB* locus in a strain lacking *murAA*. Similar to the Δ*murAA* Δ*ireB* strain, the Δ*murAA ireB* D50A strain was not inhibited by STSP (Fig. 1B and C), consistent with the model that physical interaction between IreB and MurAB restricts MurAB activity in *E. faecalis* and accounts for the lack of growth upon STSP treatment of the Δ*murAA* mutant.

**Fig 5:**
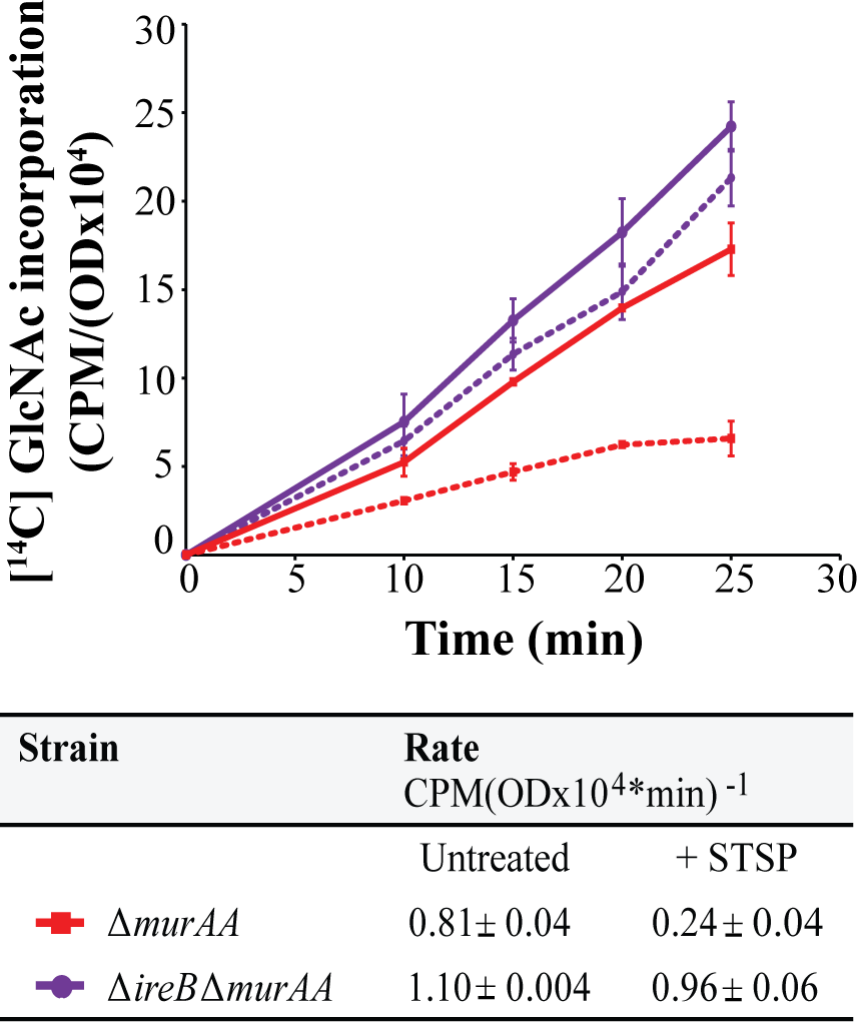
Deletion of *ireB* restores PG synthesis in the Δ*murAA* background during STSP treatment. [^14^C]GlcNAc incorporation into PG during pulse labeling of the Δ*murAA* vs Δ*ireB* Δ*murAA* strains exposed (dotted lines) or not (solid lines) to 10 µM STSP during exponential growth. Incorporation rates were determined from data points between 10 and 25 min and are reported in the table. Data represent mean ± standard deviation for two independent replicates. Strains were Δ*murAA,* JL626, and Δ*ireB* Δ*murAA,* CM32. CPM, counts per minute; OD, optical density.

To determine if IreB association with MurAB impacts PG synthesis, we monitored incorporation of [^14^C]GlcNAc into peptidoglycan (as described previously; [17, 18, 39–41]) in the presence of STSP. The [^14^C]GlcNAc incorporation rate was substantially reduced in the Δ*murAA* mutant (where all PG synthesis relies on MurAB) during STSP treatment, an effect that was largely absent in the Δ*murAA* Δ*ireB* mutant. Moreover, in the absence of STSP, the Δ*murAA* Δ*ireB* strain also incorporated [^14^C]GlcNAc at a faster rate than the Δ*murAA* strain (Fig. 5). Taken together, these observations are consistent with the model that unphosphorylated IreB inhibits the activity of MurAB *in vivo* with consequences on PG synthesis. Thus, IreB regulates both PG synthesis pathway committed-step enzymes (MurAA and MurAB) but via distinct mechanisms.

**Fig 6:**
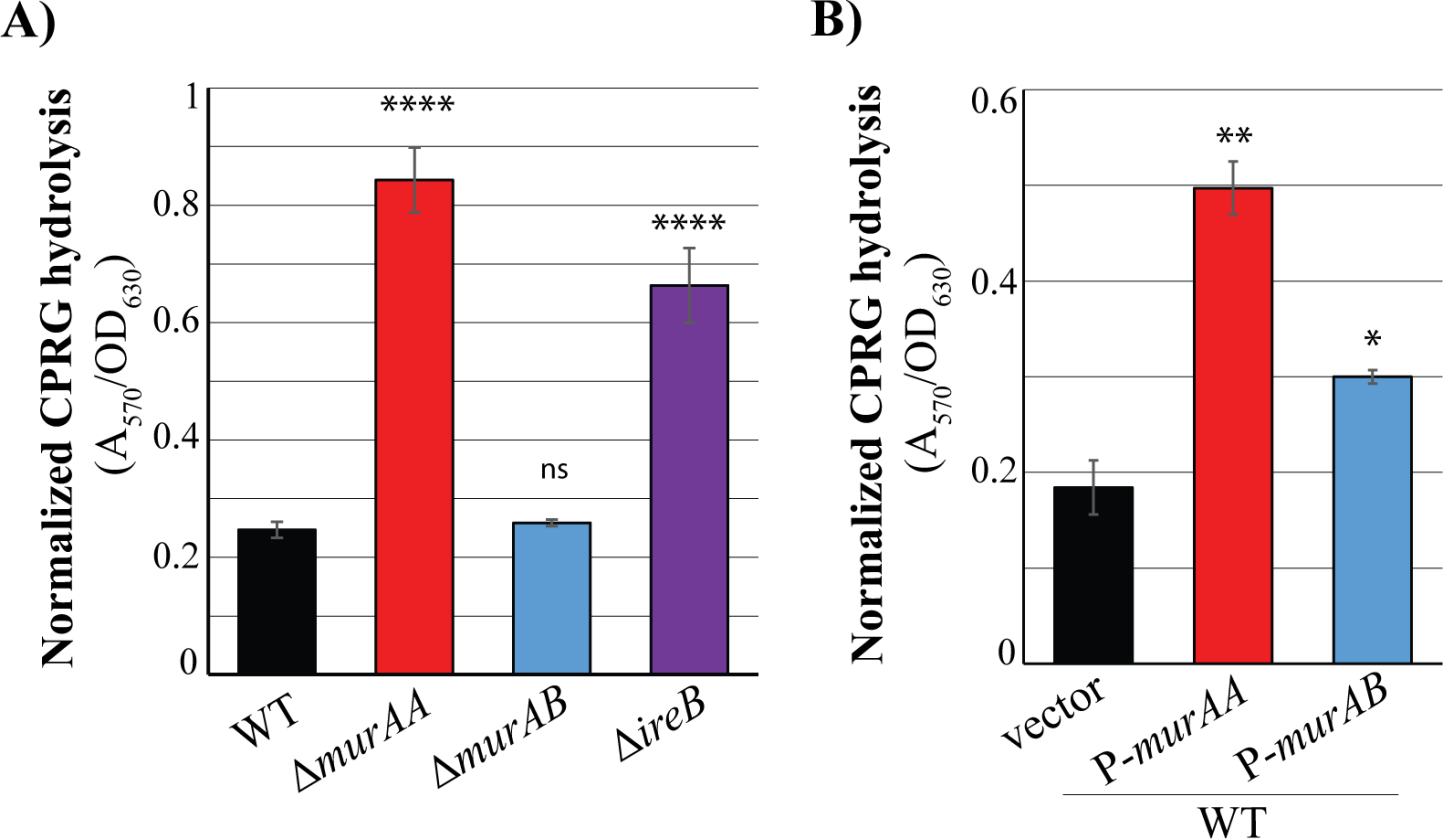
Unregulated MurAA and MurAB result in compromised cell envelope integrity. Cell envelope integrity of *E. faecalis* strains was monitored by measuring hydrolysis of CPRG after growth in Mueller-Hinton broth. (**A**) Strains used: WT, wild type, OG1; Δ*murAA*, JL626; Δ*murAB*, CM1; Δ*ireB*, JL367. Data represent the mean ± standard error from five independent biological replicates. (**B**) Strains used: WT, wild type, OG1; vector, pJRG9; *P-murAA*, pDDJ99; *P-murAB*, pJLL217; cultures were supplemented with chloramphenicol to maintain the plasmid. Data represent the mean ± standard error from three independent biological replicates. *, *P* < 0.01; **, *P* < 0.001; ****, *P* < 0.0001 determined via *t-*test relative to wild type. ns, *P* > 0.05.

To determine if IreB-mediated regulation of UNAG-CTase activity and cell wall synthesis had physiological consequences on cell wall integrity, we used a previously described assay to measure LacZ-mediated hydrolysis of chlorophenol red galactopyranoside (CPRG). CPRG is normally excluded from wild-type *E. faecalis* cells and, therefore, does not get significantly hydrolyzed by cytoplasmic LacZ. However, mutants in which the integrity of the cell wall is compromised (such as the Δ*murAA* mutant) can enable access of CPRG to LacZ and subsequent hydrolysis to yield a red product. We analyzed CPRG hydrolysis by strains exhibiting a loss of proper IreK-IreB-mediated regulation of UNAG-CTase activity (either deletion of IreB or overexpression of MurAA or MurAB), revealing that unregulated UNAG-CTase activity results in compromised integrity of the cell wall as reflected by increased CPRG hydrolysis (Fig. 6) and, moreover, that the loss of regulation of either MurAA or MurAB can account for this. Thus, collectively, these results indicate that IreB-mediated regulation of UNAG-CTase activity via both MurAA and MurAB plays an important role in control of PG synthesis to maintain cell wall integrity.

### Regulation of UNAG-CTase activity by IreB impacts competitive fitness

**Fig 7:**
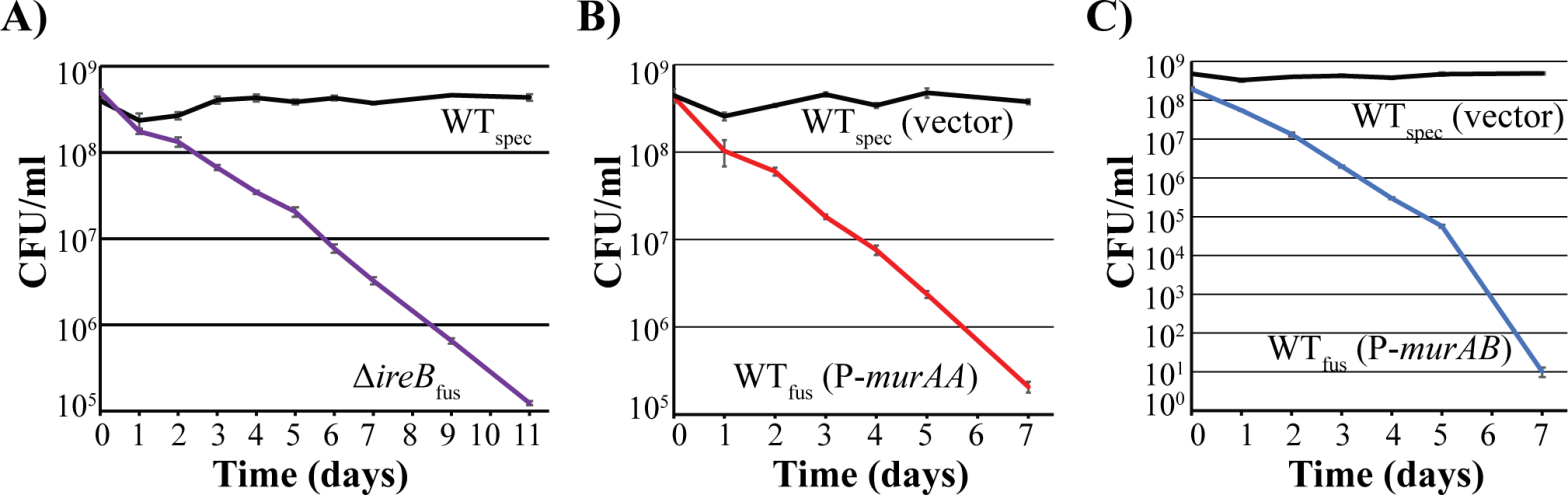
Unregulated MurAA and MurAB result in a competitive fitness defect. *E. faecalis* strains grown in Mueller-Hinton (MH) broth (chloramphenicol was supplemented when plasmid-containing strains were used) were normalized, mixed 1:1 for co-culture, and subsequently diluted into fresh MH broth. Samples were plated for CFU on indicated days on MH agar plates supplemented with spectinomycin or fusidic acid to enumerate differentially marked strains. (**A**) Co-culture of wild type (WT) with the Δ*ireB* deletion mutant. (**B**) Co-culture of wild type and the MurAA overexpression strain. (**C**) Co-culture of wild type and the MurAB overexpression strain. Data represent four biological replicates. Strains were WT_spec_, OG1Sp, spectinomycin-resistant wild type; Δ*ireB*_fus_, JL597, fusidic acid-resistant Δ*ireB* mutant; WT_fus_, CK138, fusidic acid-resistant wild type; vector, pJRG9; P-*murAA*, pDDJ99; P-*murAB*, pJLL217.

If IreB-mediated regulation of UNAG-CTase activity is a biologically meaningful means of coordinating PG synthesis, we hypothesized that perturbation of this regulatory mechanism would have consequences on cellular fitness. To test this, we performed competition experiments in which strains with altered UNAG-CTase regulation were co-cultured with wild-type cells, and the abundance of each strain in the co-culture was measured at intervals. To facilitate quantitation of the distinct strains in co-cultures, each strain was differentially marked with an unrelated antibiotic resistance marker (either fusidic acid or spectinomycin resistance). Although each strain was present at equal abundance at the start of each competition experiment, strains in which IreB-mediated regulation of UNAG-CTase activity was lost (either by deletion of IreB or by overexpression of MurAA or MurAB) were always outcompeted by wild-type cells during co-culture, reflected by the inability of strains with altered UNAG-CTase regulation to persist in the cultures (Fig. 7). This was true even for pairs of strains in which the fusidic acid and spectinomycin resistance markers were swapped (Fig. S7), confirming that the identity of the resistance markers did not influence the outcome. These results indicate that proper control of PG synthesis via IreB-mediated regulation of UNAG-CTase activity is essential to maximize competitive fitness.

## DISCUSSION

The first committed step of the peptidoglycan biosynthesis pathway is an important regulatory point in some bacteria (2, 3, 7, 13–17, 42). While the genomes of proteobacteria tend to encode only a single UNAG-CTase, low-GC gram-positive organisms in the *Firmicutes* phylum encode two homologous enzymes capable of catalyzing this reaction (5). The primary enzyme that carries out this step is regulated by proteolysis in a PASTA-kinase-dependent manner in many of the gram-positive bacteria that have been examined. This regulatory scheme was initially characterized in *L. monocytogenes* but has since been demonstrated to be intact in *B. subtilis*, *S. aureus,* and *E. faecalis* (13–17). The prevailing model from these studies is that activation of PASTA kinase signaling during cell envelope stress leads to phosphorylation of IreB (aka ReoM), thereby blocking ClpCP-mediated proteolysis of the primary UNAG-CTase to result in UNAG-CTase accumulation and, subsequently, in increased rates of peptidoglycan synthesis to strengthen the cell wall. Yet, little was previously known about the role or regulation of the secondary UNAG-CTase homolog, as it is not proteolytically regulated (at least in species where it has been examined). Furthermore, modulation of proteolysis of the primary UNAG-CTase is the only demonstrated role of the PASTA-kinase substrate IreB/ReoM in any organism to date. Our results reveal that in *E. faecalis,* the IreK-IreB signaling axis also directly regulates the enzymatic activity of the secondary UNAG-CTase homolog (MurAB) through phosphorylation-modulated physical interaction between IreB and MurAB. Hence, our work establishes not only a new regulatory target and mechanism of action for IreB but also the first described regulatory mechanism for a MurAB homolog in any organism.

Unphosphorylated IreB from *E. faecalis* can directly interact with either MurAA or MurAB. The consequences of these interactions are distinct: the targeted proteolysis of MurAA by the ClpCP protease (17) and, as reported here, direct inhibition of MurAB enzymatic activity (Fig. S8). During unstressed exponential growth of *E. faecalis*, the cellular pool of IreB is partially phosphorylated (28), suggesting that the activities of MurAA and MurAB might be partially restricted by the remaining unphosphorylated IreB during unstressed exponential growth. Upon activation of IreK in response to cell envelope stress, the pool of IreB becomes more phosphorylated, which would prevent physical interaction between IreB and MurAA or MurAB, thereby allowing for a boost in PG synthesis to overcome the stress. Thus, we speculate that unstressed but growing *E. faecalis* cells possess excess capacity for PG synthesis that remains “in reserve” until it is called for when the cell encounters cell wall-damaging stressors, at which time the IreK-IreB signaling axis can mobilize this excess PG-synthesizing capacity by unleashing MurAA and MurAB as part of the adaptive biological response. The concept that cells might hold some PG synthesis capacity in reserve has a precedent: Sun and co-workers previously reported that *B. subtilis* restricts its growth under nutrient-limiting conditions, leading them to hypothesize that *B. subtilis* retains a “reserve” growth capacity under certain stress conditions that allows for fine-tuning in response to different stimuli (16). In *E. faecalis*, IreB-mediated regulation of UNAG-CTase activity appears to be biologically critical: not only does loss of this control lead to elevated PG synthesis (Fig. 5), but it also compromises the integrity of the cell wall (Fig. 6) and impairs competitive fitness (Fig. 7). Thus, “too much” or dysregulated PG synthesis is not necessarily beneficial. This concept of harboring excess PG-synthesizing capacity “in reserve” might also suggest an explanation for why gram-positive bacteria retain two UNAG-CTases in their genomes: these bacteria are known to produce much more PG per cell than gram-negative bacteria such as *E. coli*, and perhaps a single UNAG-CTase might not be sufficient to meet the enhanced demand for PG that occurs when gram-positive cells encounter antimicrobials that impair PG synthesis. In such a scenario, the “instantaneous” release of MurAB inhibition that occurs upon IreK-mediated phosphorylation of IreB may enable a burst of PG synthesis to meet the immediate needs of the cell while waiting for the primary UNAG-CTase MurAA to accumulate through new translation, thereby maximizing overall cellular fitness.

IreB exists as a dimer both *in vitro* and *in vivo* (34), and AlphaFold3 modeling suggests that each subunit of the IreB dimer can interact with a monomer of MurAB (Fig. 3). Because IreB can also interact with MurAA (17) (modeled in Fig. S9A), we speculate that the IreB dimer might be capable of binding a monomer of both MurAA and MurAB simultaneously. In support of such a hypothesis, AlphaFold3 modeling (35) returned a high-confidence prediction for a MurAA-2IreB-MurAB complex (Fig. S9B). Such a complex would be consistent with the model that IreB coordinately regulates these two enzymes. An alternative partner-switching scenario in which MurAA and MurAB “compete” for binding to IreB seems less likely, primarily due to our earlier observation that deletion of MurAB from *E. faecalis* leads to protection of MurAA from proteolysis (17) (the outcome that occurs when IreB cannot bind MurAA). If competition for IreB between MurAA and MurAB was occurring, the absence of MurAB might be expected to enhance the IreB-MurAA interaction and, therefore, presumably increase overall proteolysis of MurAA. Instead, we speculate that stabilization of MurAA in *E. faecalis* upon loss of MurAB suggests that formation of a MurAA-2IreB-MurAB heterotetrameric complex may be required for MurAA proteolysis *in vivo*.

Studies in *S. pneumoniae* suggest that the direct regulation of UNAG-CTase enzymatic activity by IreB homologs may be a widespread phenomenon. The UNAG-CTase homologs (MurA or MurZ) in *S. pneumoniae* are not regulated by proteolysis*,* yet increasing MurA or MurZ levels is sufficient to overcome defects associated with impaired signaling by the pneumococcal PASTA kinase, StkP, suggesting that MurA and/or MurZ are downstream of StkP/IreB signaling in some way (7). Thus, IreB may directly regulate the enzymatic activity of one or both of the UNAG-CTases in *S. pneumoniae* in a manner analogous to MurAB of *E. faecalis*. If so, *E. faecalis* might possess a “hybrid” IreB-dependent regulatory scheme that encompasses both proteolytic regulation of MurAA (shared with several other *Firmicutes* like *L. monocytogenes, S. aureus,* and *B. subtilis*) and direct enzymatic regulation of MurAB (potentially shared with *S. pneumoniae* and others).

In conjunction with our earlier work (17), the results presented here demonstrate a mechanism for coordinated but distinct regulation of the two UNAG-CTases in *E. faecalis* by a single PASTA kinase substrate (IreB). This work underscores the central role of IreB and PASTA kinase signaling in resource allocation for peptidoglycan synthesis and furthers our collective understanding of the control of this critical biosynthetic pathway.

## MATERIALS AND METHODS

### Bacterial strains and growth conditions

Bacterial strains and plasmids used in this study are listed in Table S1. Enterococci were propagated on Mueller-Hinton broth (MHB) or agar (prepared according to the manufacturer’s instructions; Difco) at 30°C unless indicated otherwise. *E. coli* strains were grown in lysogeny broth (LB) or agar. Both TOP10 and DH5α were used as routine cloning hosts. Antibiotics were included, when necessary, for maintenance of plasmids at the following concentrations: chloramphenicol, 20 µg mL^−1^ (*E. coli*) or 10 µg mL^−1^ (*E. faecalis*); kanamycin, 50 µg mL^−1^; ampicillin, 100 µg mL^−1^; erythromycin, 100 µg mL^−1^ (*E. coli*) or 10 µg mL^−1^ (*E. faecalis*).

### Generation of plasmids

Recombinant plasmids were generated using isothermal assembly (43), and inserts were sequenced in their entirety to ensure the absence of any undesired mutations.

The *ireB* D50A allele used to generate pCAM113 (pKNT25) and pCAM114 (pUT18c) for bacterial two-hybrid analysis and the pET28a-based plasmid for purification of recombinant IreB D50A (pCAM143) was amplified from strain JG4 (32). All other plasmids were generated by amplifying wild-type alleles from *E. faecalis* OG1RF genomic DNA.

### Genetic manipulation of enterococci

Mutant strains were generated using the temperature-sensitive, counterselectable allelic exchange plasmid pJH086 as described previously (44). Counterselection plates contained 20% sucrose and p-Cl-Phe. Deletion alleles were generated in-frame. Fragments of genomic DNA upstream and downstream of the gene of interest, which included a small number of codons at the beginning and end of each gene, were amplified and introduced into pJH086 by isothermal assembly (43). All mutant or complemented strains were constructed independently at least twice and analyzed to ensure that their phenotypes were concordant.

The *ireB* D50A allele was amplified from strain JG4 to generate the pJH086-based plasmid, pCAM133, which was then introduced into OG1 and JL626 to generate strains CM70 (*ireB* D50A) and CM71 (*ireB* D50A Δ*murAA*), respectively.

Strain CM32 was generated by deleting the *murAA* allele from JL367 using pJLL229. CM62 was generated by deleting *clpC* from JL626 using pCAM70.

Strain DDJ470 (pJLL288) was generated by deleting the *murAA* allele from JL206 using pJLL229 in the presence of 5 mM NaNO_3_ inducer.

### Analysis of growth ± STSP

Stationary-phase *E. faecalis* strains were diluted to a normalized density of OD_600_ = 4 × 10^−5^ (~1 × 10^5^ CFU) in MHB supplemented with a final concentration of 10 µM staurosporine (Millipore) resuspended in DMSO, or equivalent volume DMSO (untreated) and chloramphenicol for plasmid maintenance as needed in a 100-well honeycomb plate. Plates were incubated in a Bioscreen C plate reader at 37℃ for 24 h with brief shaking before each measurement. The optical density at 600 nm (OD_600_) was determined every 15 min. At least three independent replicates were analyzed for each strain.

### Protein purification

Recombinant MurAB-His_6_ and MurAA-His_6_ were purified from *E. coli* C43(DE3) and BL21(DE3) cells, respectively, as described previously (17).

His_6_-SUMO-IreB was expressed and purified from *E. coli* BL21(DE3) cells as described previously (17) with the following exceptions. Wild-type IreB used in Fig. 2 and Fig. S5A was purified using 50 mM 3-(*N*-morpholino)propanesulfonic acid (MOPS), 350 mM NaCl (pH = 6.6) buffer because the MurAB/MurAA enzymatic assay is incompatible with a phosphate-based buffer. The MOPS buffer was supplemented with 20 mM imidazole for wash steps during affinity chromatography and 250 mM imidazole for elution of His_6_-SUMO-IreB from the Ni-charged resin. His_6_-SUMO-IreB D50A was also purified using the MOPS buffer but otherwise proceeded as previously described for wild-type IreB.

His_6_-IreK-n (intracellular domain only) used for *in vitro* phosphorylation of recombinant IreB was purified as described previously (30).

### UDP-N-acetylglucosamine 1-carboxyvinyl transferase activity assays

Enzymatic activity of purified MurAB and MurAA was determined by measuring release of inorganic phosphate (P_i_) as described previously with modifications (9, 17). In a 100-µL final reaction volume, 0.1 uM MurAA or MurAB was pre-incubated with 20 mM UDP-GlcNAc and 1 µM bovine serum albumin (BSA) in the presence or absence of 1 µM IreB as indicated (or equivalent volume buffer) in 50 mM Tris, 2 mM dithiothreitol (DTT) (pH = 7.5) for 15 min at room temperature. Phosphoenolpyruvate (1 mM) was added to initiate the reactions which proceeded at room temperature. Control reactions to measure background signal were set up as described above but without the addition of UDP-GlcNAc. At intervals, aliquots were removed and assayed using the molybdate dye component from the Ser/Thr Phosphatase Assay System Kit (Promega). Color development was measured 12 min after each time point compared to a P_i_ standard curve. Replicate measurements were made as indicated in the figure legend, and measurements were background adjusted using no-UDP-GlcNAc control reactions.

For experiments analyzing the effect of phosphorylated IreB on MurAB activity, IreB was phosphorylated *in vitro* as previously described (17, 30). Briefly, 100 µM IreB was incubated with 1 µM purified IreK-n (kinase domain) in the presence of 5 mM MgCl_2_ in kinase buffer (50 mM Tris, 25 mM NaCl [pH = 7.5]). ATP (2 mM) was added to start the reaction, which proceeded for 40 min at 37°C. The phosphorylation reactions were then subjected to buffer exchange using Zeba Spin Desalting columns (Thermo Fisher Scientific) into kinase buffer to remove excess ATP and MgCl_2_ prior to use in the MurAB enzymatic assay. Two microliters of the buffer-exchanged kinase reaction (or control reactions containing IreB alone [i.e., unphosphorylated IreB] or IreK alone) was then used for the analysis of MurAB activity. The final concentration of IreB in these reactions is expected to be ≤2 µM. IreK is also expected to be present at ≤0.02 µM but was not found to impact MurAB activity at this concentration (Fig. S5). Measurement of MurAB activity proceeded as described above.

### AlphaFold3 modeling

Protein-protein interaction modeling was performed using Google’s AlphaFold Server as described in reference 35. The top-ranked model was visualized and annotated using UCSF ChimeraX (45).

### Bacterial two hybrid

Experiments were performed as described previously (17). Briefly, *E. faecalis* genes were cloned as fusions to the T18 and T25 domains of *B. pertussis* adenylate cyclase and co-electroporated into adenylate-cyclase-deficient *E. coli* DHM1 cells. Strains were grown to stationary phase in LB supplemented with 50 µg mL^−1^ kanamycin, 100 µg mL^−1^ ampicillin, and 0.1 mM IPTG, then spotted on agar plates of the same media also containing 150 µg mL^−1^ X-gal. Spot plates were incubated for 5 days at room temperature, and three independent co-transformants were analyzed for color development for each combination. The leucine zipper domains of GCN4 (zip) were used as non-interacting controls.

### Thermal shift assays

Protocol was adapted from reference 37 and described in greater detail in reference 17. Briefly, 2.5 µM purified, recombinant MurAB was combined with either IreB, IreB D50A, or lysozyme (control protein; Sigma-Aldrich) at the indicated ratios in the presence of a final concentration of 10× SYPRO Orange dye (Invitrogen; 5,000× concentrate in DMSO diluted to 100× in water before use) in 50 mM Tris, 25 mM NaCl (pH = 7.5). MurAB samples, in triplicate, were incrementally heated to 95°C, and fluorescence emission was measured as a function of temperature. The thermal shift for MurAB (in °C) was reported for each molar ratio of IreB/IreB D50A/lysozyme tested relative to MurAB alone.

### Peptidoglycan synthesis assays

Incorporation of [^14^C]GlcNAc into SDS-insoluble peptidoglycan was monitored as described previously with modifications (17, 18, 39–41). Briefly, cells were grown to exponential phase in MHB at 37°C, then treated ±10 µM staurosporine, and incubated for an additional 10 min at 37°C. Following pre-treatment with STSP, cells were diluted sixfold into pre-warmed media containing 0.33 µCi mL^−1^ [^14^C]GlcNAc (PerkinElmer) with and without STSP and incubated at 37°C. At intervals, aliquots were mixed with equal volume of 0.2% SDS, pelleted, and radioactivity in the SDS-insoluble pellets was measured using scintillation counting and normalized to optical density measurements taken in triplicate for parallel unlabeled cultures.

### CPRG hydrolysis assay

CPRG hydrolysis assay was done as described previously (46) with minor modifications. Cultures were grown to stationary phase at 37°C in MHB supplemented with 10 µg/mL erythromycin (for maintenance of pCJK205) and 40 µg/mL chlorophenol red-β-D-galactopyranoside; chloramphenicol was supplemented in the cultures when MurAA and MurAB overexpression plasmids were used. CPRG hydrolysis was quantified by measuring absorbance of cell-free supernatants at 570 nm and normalizing to cell density measured by optical density at 630 nm.

### Co-culture competition assay

Stationary-phase cultures of *E. faecalis* strains were normalized and mixed 1:1 as indicated in the legends using four independent biological replicates of each culture. Tenfold serial dilutions of the mixed cultures were then prepared and plated for CFU enumeration on (i) MHB supplemented with spectinomycin 500 µg/mL to enumerate OG1Sp-derived strains and (ii) MHB supplemented with 25 µg/mL fusidic acid to enumerate the CK138-derived strains. When strains contained a plasmid, chloramphenicol was supplemented in the cultures and agar plates at 10 µg/mL. The co-cultures were diluted 1 × 10^4^-fold in MHB and incubated at 37°C, overnight, 225 rpm. The mixed cultures were diluted and incubated each day and enumerated as described above.

## ADDENDUM

The IreB homolog from *Streptococcus pneumoniae* has recently been shown to directly inhibit UNAG-CTase enzymatic activity *in vitro* as well (M. Winkler, personal communication).

## SUPPLEMENTAL MATERIAL

Supplemental Material mbio.00593-25-s0001.pdf Figures S1 to S9, supplemental methods, and Table S1.

ASM does not own the copyrights to Supplemental Material that may be linked to, or accessed through, an article. The authors have granted ASM a non-exclusive, world-wide license to publish the Supplemental Material files. Please contact the corresponding author directly for reuse.
