# Supplementary material for: PASTA kinase signaling regulates peptidoglycan synthesis in Enterococcus faecalis by direct inhibition of UDP-N-acetylglucosamine 1-carboxyvinyl transferase activity
Source: mBio. 2025 Apr 24;16(5):e00593-25. doi: 10.1128/mbio.00593-25 (PMC12077105; doi:10.1128/mbio.00593-25)
Supplement: Supplemental Material — Figures S1 to S9, supplemental methods, and Table S1. [file mbio.00593-25-s0001.pdf]

Supplemental material for:

PASTA kinase signaling regulates peptidoglycan synthesis in *Enterococcus faecalis* by direct inhibition of UDP-N-acetylglucosamine 1-carboxyvinyl transferase activity

Authors: Carly A. Mascari[1], Dušanka Djorić[1], and Christopher J. Kristich*

[1]These authors contributed equally.

Affiliation:

Department of Microbiology and Immunology

Center for Infectious Disease Research

Medical College of Wisconsin

8701 Watertown Plank Rd

Milwaukee, WI 53226

*For correspondence: ckristich@mcw.edu

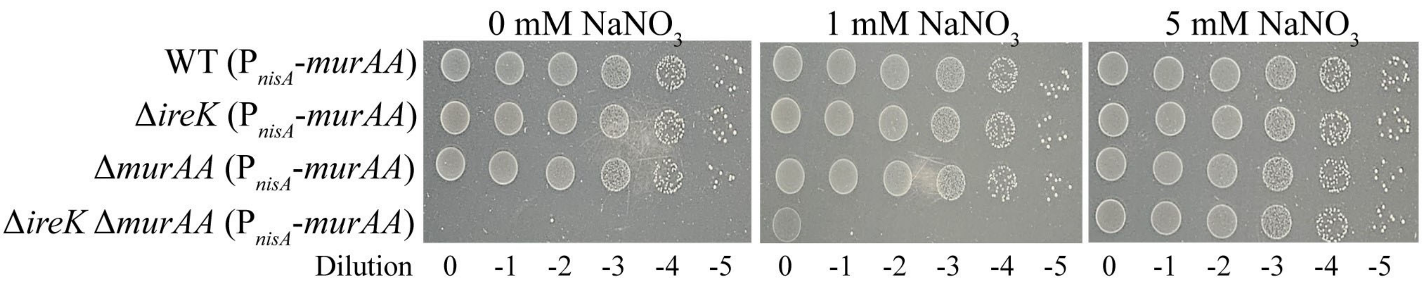

Figure S1 | *murAA* and *ireK* are synthetically lethal. Cultures grown in the presence of 5 mM $NaNO_3$ were washed, subjected to 10-fold serial dilutions, and spotted on MHB supplemented as indicated and incubated at 37 °C for 24 h. Results are representative of a minimum of 2 independent biological replicates. Strains were Wild-type/ P_{nisA}-*murAA*, OG1/pJLL288; Δ*ireK*/ P_{nisA}-*murAA*, JL206/pJLL288; Δ*murAA*/ P_{nisA}-*murAA*, JL626/pJLL288; and Δ*ireK* Δ*murAA*/P_{nisA}-*murAA*, DDJ470/pJLL288.

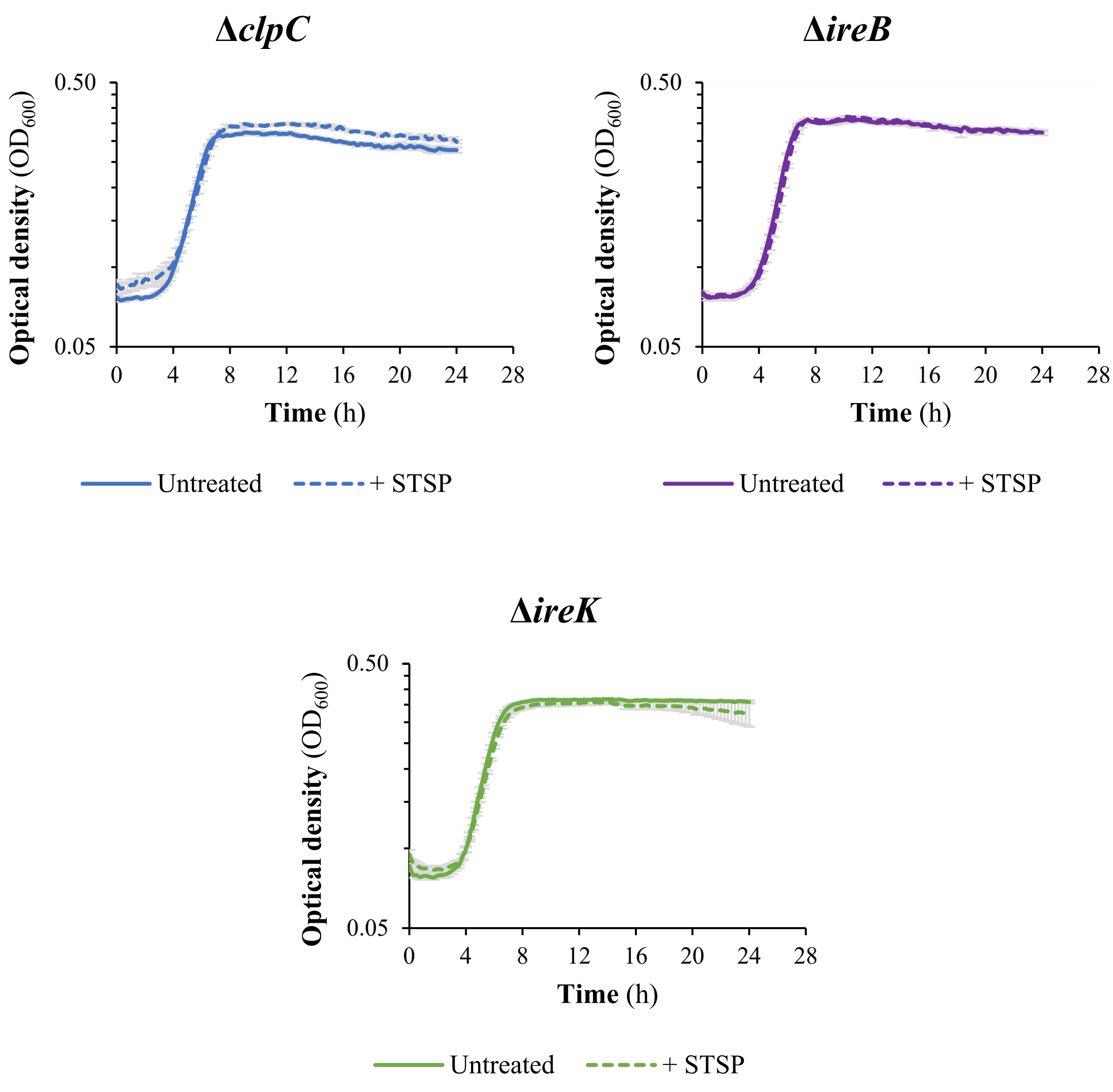

Figure S2 | Staurosporine (STSP) does not alter growth of the Δ*ireK*, Δ*clpC*, or **Δ*ireB* mutants**. Growth curves for the indicated *E. faecalis* strains grown at 37 °C with 10 µM STSP or equivalent volume DMSO (untreated) as a vehicle control. Growth curves represent an average of 3 independent replicates within each experiment and error bars show standard deviation (too small to see in many cases). Strains were Δ*ireK*, JL206; Δ*ireB*, JL367, Δ*clpC,* CM35.

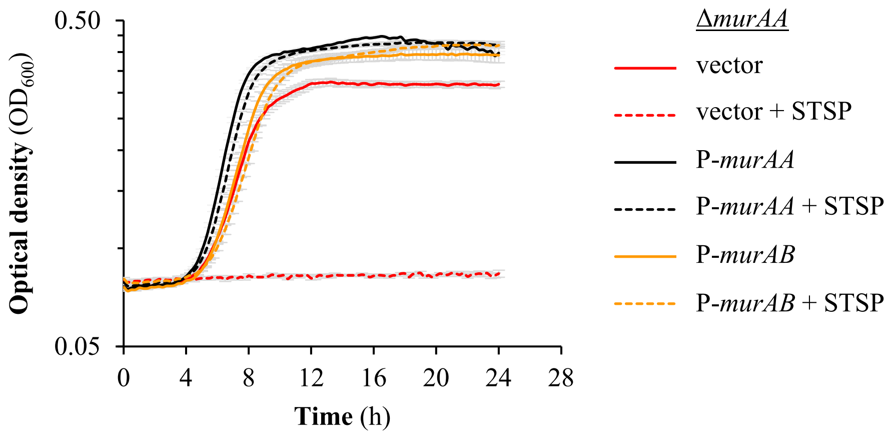

Figure S3 | Overexpression of *murAA* or *murAB* restores growth of the *murAA* deletion strain in the presence of staurosporine. Growth curves for the indicated *E. faecalis* strains grown at 37 °C with 10 µM STSP or equivalent volume DMSO as a vehicle control. Growth curves depict the average of 3 independent replicates with error bars indicating standard deviation. Strains were Δ*murAA*/vector, JL626/pJRG9; Δ*murAA*/P-*murAA*, JL626/pDDJ99 and Δ*murAA*/P-*murAB*, JL626/pJLL217.

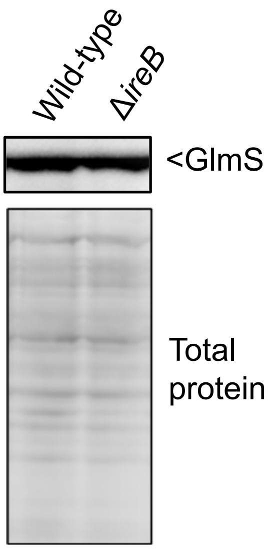

Figure S4 | Loss of IreB does not alter the steady-state abundance of GlmS. Immunoblot analysis on total cell lysates of wild-type and Δ*ireB* strains of *E. faecalis* collected during exponential phase. Blots were probed with custom anti-serum to GlmS followed by an HRP-conjugated secondary antibody. NoStain reagent was used for detection of total protein. Immunoblot is representative of three independent biological replicates. Strains are wild-type, OG1; Δ*ireB*, JL367.

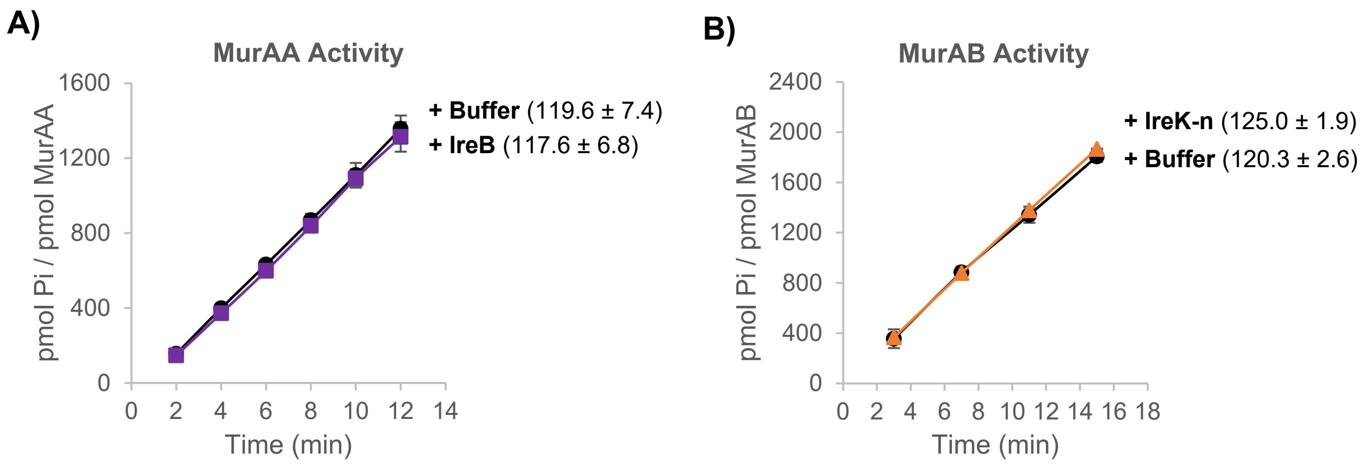

Figure S5 | (A) IreB does not inhibit enzymatic activity of MurAA. UNAG-CTase activity of purified, recombinant MurAA was measured in the presence of 10-fold molar excess purified wild-type IreB (purple squares) or equivalent volume buffer (black circles). Data represent the average of 6 replicate reactions ± standard deviation. The reaction rate was calculated as the average slope for data points between 2 and 12 min and is reported in parentheses. **(B) IreK kinase domain used for IreB phosphorylation does not impact MurAB activity.** UNAG-CTase activity of purified, recombinant MurAB was measured in the presence of IreK kinase domain at the concentration used for *in vitro* phosphorylation of IreB (IreK-n; orange triangles) or buffer (black circles). Data represent the average of 3 replicates ± standard deviation. The reaction rate was calculated as the average slope for data points between 3 and 15 min and is reported in parentheses.

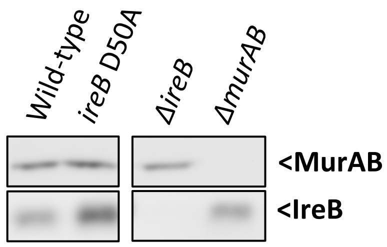

Figure S6 | The IreB D50A variant is expressed in *E. faecalis*. Immunoblot analysis was performed on lysates collected during exponential phase for the indicated strains using anti-sera to MurAB and IreB followed by an Alexa Fluor 647-conjugated secondary antibody. Blot is representative of two biological replicates. The left and right sections for each represent a single image from which intervening lanes that were not relevant to this analysis were removed for clarity. Strains were wild-type, OG1; *ireB* D50A, CM70; Δ*ireB*, JL367; Δ*murAB*, CM1.

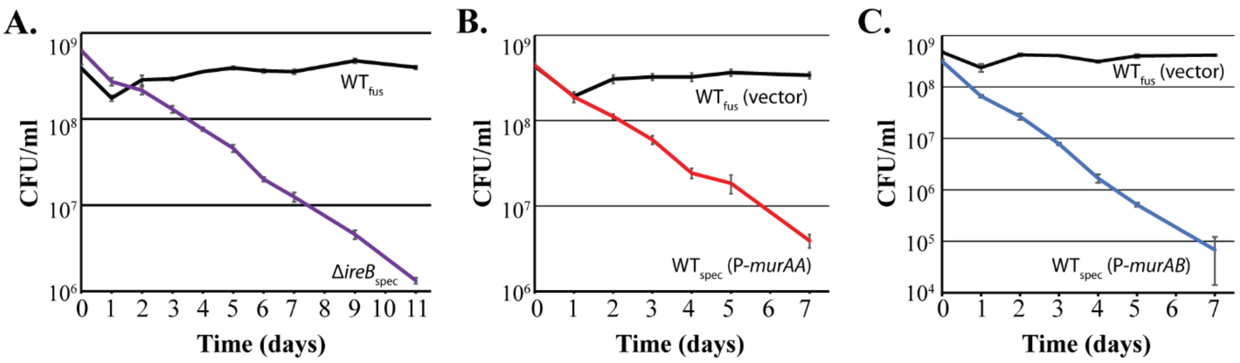

Figure S7 The fitness defect resulting from unregulated MurAA and MurAB is not due to the antibiotic marker. *E. faecalis* strains grown in MH broth (chloramphenicol was supplemented when plasmid containing strains were used) were normalized, mixed 1:1 for co-culture and subsequently diluted into fresh MH broth. Samples were plated for CFU (at Day 0 through Day 11 for panel A, and Day 0 through Day 7 for panels B and C) on MH agar plates supplemented with spectinomycin or fusidic acid to enumerate differentially marked strains. (A) Co-culture of wild-type (WT) with the Δ*ireB* deletion mutant. (B) Co-culture of wild-type and the MurAA overexpression strain. (C) Co-culture of wild-type and the MurAB overexpression strain. Data represents four biological replicates. Strains were: WT_{fus}, CK138, fusidic acid resistant wild-type; $\Delta ireB_{spec}$, JL598, spectinomycin resistant Δ*ireB* mutant; WT*spec*,OG1Sp, spectinomycin resistant wild-type; vector, pJRG9; P-*murAA*, pDDJ99; P-*murAB*, pJLL217.

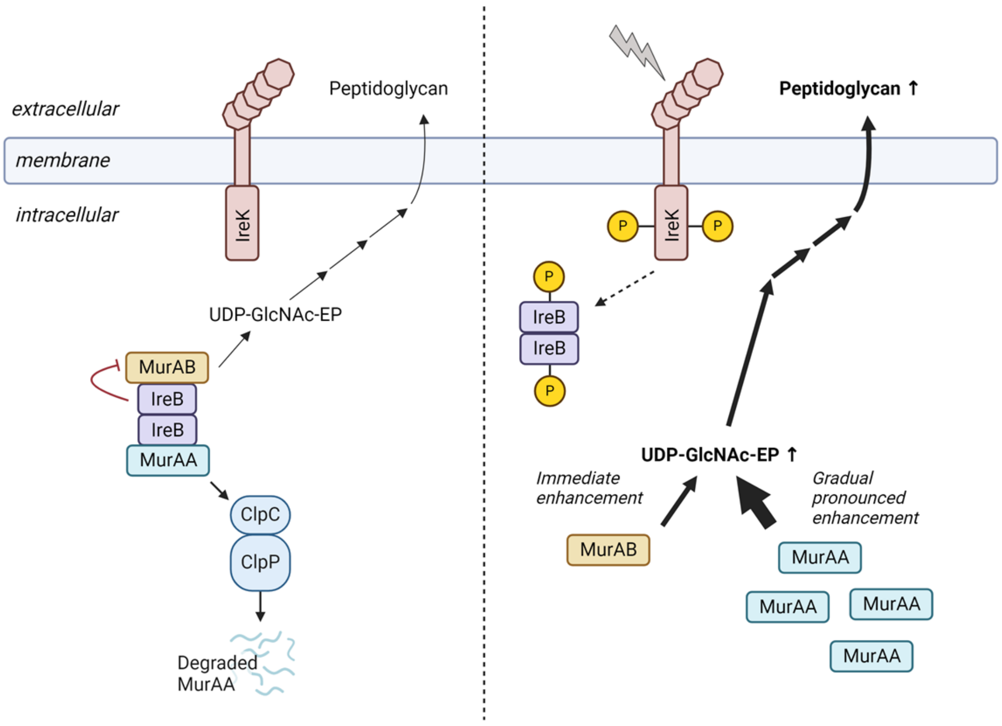

Figure S8 | Model in which IreB provides coordinated, but differential regulation of MurAA and MurAB. When IreK is not activated, IreB remains unphosphorylated and physically interacts with both MurAA and MurAB simultaneously. Under these conditions MurAA is targeted for degradation by the ClpCP complex and accordingly its cellular abundance is low. Meanwhile, the enzymatic activity of MurAB is inhibited by IreB. Thus, synthesis of UDP-N-acetylglucosamine enolpyruvate (UDP-GlcNAc-EP), and subsequently entry of substrates into the peptidoglycan synthesis pathway, is restricted. Following activation of IreK, such as during exposure to cell envelope stress, IreB becomes phosphorylated by IreK and is no longer competent to interact with either MurAA or MurAB. This results in an immediate enhancement of the enzymatic activity of MurAB, providing a modest increase in synthesis of UDP-GlcNAc-EP. MurAA also gradually begins to accumulate in cells given that is no longer targeted for degradation by ClpCP. Accumulation of MurAA appears to result in a more dramatic increase in peptidoglycan synthesis and has consequences of growth and resistance to cephalosporin antibiotics.

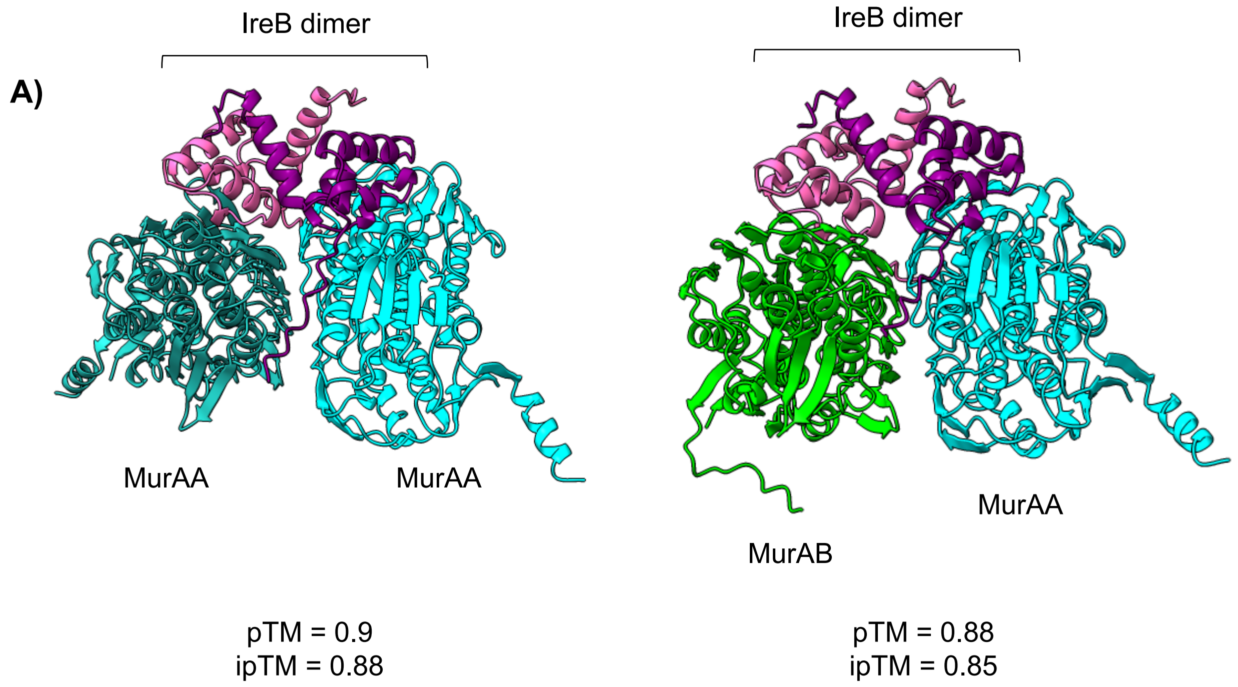

Figure S9 | AlphaFold3 modeling. Prediction complexes between (A) two monomers of MurAA (cyan and teal; WP_002356562.1) and an IreB homodimer (purple and pink; WP_002357937.1) and (B) the IreB homodimer and one monomer each of MurAA (cyan; WP_002356562.1) and MurAB (light green; WP_002357969.1).

SUPPLEMENTAL EXPERIMENTAL PROCEDURES

Agar-plate growth assays for depletion strain. Overnight cultures grown in MH broth supplemented with 10 µg/ml erythromycin and 5 mM $NaNO_3$ for induction of plasmid-borne *murAA* expression were centrifuged. The pellets were washed 2x with MH broth to remove residual $NaNO_3$ prior to normalization. Ten-fold serial dilutions were then prepared and 4 µl spotted on MH agar plates supplemented with 10 µg/ml erythromycin and $NaNO_3$ as indicated.

Immunoblot analysis. Stationary-phase cultures were diluted to $OD_{600} = 0.01$ in MHB and incubated at 37 °C with shaking. Cultures were grown until OD_{600} ~0.2 and harvested by mixing with an equal volume of cold EtOH/acetone (1:1). After harvesting, cells were pelleted by centrifugation and washed once with water. To prepare total cell lysates, bacteria were suspended in lysozyme buffer (10 mM Tris, 50 mM NaCl, 20% sucrose [pH 8]), normalized to equivalent OD_{600}, treated with lysozyme (5 mg ml^{-1}) for 30 min at 37°C, mixed with Laemmli SDS sample buffer containing 100 mM DTT and boiled for 5 min. After SDS-PAGE, proteins were transferred to polyvinylidene difluoride (PVDF; Bio-Rad) using a Trans-blot Turbo system (Bio-Rad) and incubated with No-Stain Protein Labeling reagent (Invitrogen) when indicated for visualization of total protein prior to blocking with 5% skim milk in Tris-buffered saline. Membranes were then probed with custom rabbit anti-GlmS, MurAB or IreB polyclonal antiserum followed by either goat anti-rabbit, Alexa Fluor 647-conjugated secondary antibody (Invitrogen) or goat anti-rabbit horseradish peroxidase-conjugated secondary antibody (Invitrogen) as indicated in the figure legend. Imaging of membranes was performed using an Amersham Typhoon 5 (Cytiva, Marlborough, MA) for Alexa Fluor 647-conjugated secondary antibody and No-Stain reagent or using Super-Signal (Thermo Fisher Scientific, Waltham, MA) on a ChemiDoc Touch System (Bio-Rad, Hercules, CA) for HRP-conjugated secondary antibody.

Table S1 | Plasmids and strains used in this work

Strain or plasmid	Relevant genotype or description[a]	Source or reference
**Strains**		
E. coli		
DH5α	routine cloning host	Lab stock
TOP10	routine cloning host	Lab stock
DHM1	*cya*$^{-}$ *rec*$^{-}$ reporter strain for protein-protein interaction detection	Lab stock
BL21 (DE3)	Protein overexpression host	Lab stock
C43 (DE3)	Protein overexpression host optimized for toxic proteins	Lucigen
E. faecalis		
OG1	Wild-type reference strain (MLST 1)	(1)
JL626	OG1 Δ*murAA*	(2)
JL367	OG1 Δ*ireB*	(3)
CM32	OG1 Δ*ireB* Δ*murAA*	This work
CM62	OG1 Δ*murAA* Δ*clpC*	This work
CM70	OG1 *ireB* D50A	This work
CM71	OG1 *ireB* D50A Δ*murAA*	This work
CM1	OG1 Δ*murAB*	(2)
OG1RF	Wild-type reference strain (MLST 1)	(4)
JG4	Ceftriaxone-resistant suppressor mutant of OG1RF Δ*ireK*; carries D50A mutation in *ireB*	(5)
DDJ470 (pJLL288)	OG1 Δ*ireK* Δ*murAA* (P_{nisA}-*murAA*)	This work
OG1Sp	Spontaneous Sp^{R} derivative of OG1	(6)
CK138	Spontaneous Fa^{R} derivative of OG1	(7)
JL597	CK138 Δ*ireB*	This work
JL598	OG1Sp Δ*ireB*	This work
**Plasmids**		
pJH086	*E. faecalis* allelic exchange vector (Cm^{R}); *pheS** counterselection	(8)
pJLL229	pJH086 carrying Δ*murAA* allele, used for generation of CM32	(2)
pCJK218	*E. faecalis* allelic exchange vector (Cm^{R}); *pheS** counterselection	(9)
pJLL73	pCJK218 carrying Δ*ireB* (ΔE6-H84)	(3)
pCAM70	pJH086 carrying Δ*clpC* allele, used for generation of CM62	(10)
pCAM133	pJH086 carrying *ireB* D50A allele amplified from JG4; used for generation of CM70 and CM71	This work

pJRG9	*E. faecalis* expression vector, constitutive $P23_s$ promoter (Cm^R)	(11)
pDDJ99	pJRG9::*murAA*-strep	(2)
pJLL217	pJRG9::*murAB*-strep	This work
pJLL286	Nitrate-inducible expression vector (Em^R)	(2)
pJLL288	pJLL286 carrying P_{nisA}::*murAA*	(2)
pKT25	*E. coli* vector used to generate fusions with the C-terminus of *cya* T25 (Kn^R)	Lab stock
pCAM73	pKT25::*murAB*	(10)
T25-zip	pKT25::GCN4 leucine zipper	Lab stock
pKNT25	*E. coli* vector used to generate fusions with the N-terminus of *cya* T25 (Kn^R)	Lab stock
pSLK148	pKNT25::*ireB*	(10)
pCAM113	pKNT25::*ireB* D50A	This work
pUT18c	*E. coli* vector used to generate fusions with the C-terminus of *cya* T18 (Amp^R)	Lab stock
pSLK163	pUT18c::*ireB*	(10)
pCAM114	pUT18c::*ireB* D50A	This work
pCAM72	pUT18c::*murAB*	(10)
T18-zip	pUT18c::GCN4 leucine zipper	Lab stock
pET28b	*E. coli* expression vector (Kn^R)	Novagen
pDV20	pET28b::*murAA*-His_6	(12)
pDV16	pET28b::*murAB*-His_6	(10)
pCJK111	pET28b::His_6-*ireK-n*	(7)
pET28a::His_6-smt_3	*E. coli* protein expression vector (Kn^R) with cleavable His_6 SUMO tag	(13)
pCLH194	pET28a::His_6-smt_3-*ireB*	(14)
pCAM143	pET28a::His_6-smt_3-*ireB* D50A	This work
pCJK205	Constitutive expression of *lacZ* (Em^R)	(15)

[a] MLST, multilocus sequence type

SUPPLEMENTAL REFERENCES

1. O. G. Gold, H. V. Jordan, J. van Houte, The prevalence of enterococci in the human mouth and their pathogenicity in animal models. *Arch Oral Biol* **20**, 473-477 (1975).
2. C. A. Mascari, D. Djorić, J. L. Little, C. J. Kristich, Use of an Interspecies Chimeric Receptor for Inducible Gene Expression Reveals that Metabolic Flux through the Peptidoglycan Biosynthesis Pathway is an Important Driver of Cephalosporin Resistance in *Enterococcus faecalis*. *J Bacteriol* **204**, jb.00602-00621 (2022).
3. B. D. Labbe, C. J. Kristich, Growth- and Stress-Induced PASTA Kinase Phosphorylation in *Enterococcus faecalis*. *J Bacteriol* **199**, e00363-00317 (2017).
4. G. M. Dunny, B. L. Brown, D. B. Clewell, Induced cell aggregation and mating in Streptococcus faecalis: evidence for a bacterial sex pheromone. *Proc Natl Acad Sci U S A* **75**, 3479-3483 (1978).
5. C. L. Hall, M. Tschannen, E. A. Worthey, C. J. Kristich, IreB, a Ser/Thr kinase substrate, influences antimicrobial resistance in *Enterococcus faecalis*. *Antimicrob Agents Chemother* **57**, 6179-6186 (2013).
6. C. J. Kristich, J. R. Chandler, G. M. Dunny, Development of a host-genotype-independent counterselectable marker and a high-frequency conjugative delivery system and their use in genetic analysis of *Enterococcus faecalis*. *Plasmid* **57**, 131-144 (2007).
7. C. J. Kristich, J. L. Little, C. L. Hall, J. S. Hoff, Reciprocal regulation of cephalosporin resistance in *Enterococcus faecalis*. *mBio* **2**, e00199-00111 (2011).
8. S. L. Kellogg, J. L. Little, J. S. Hoff, C. J. Kristich, Requirement of the CroRS Two-Component System for Resistance to Cell Wall-Targeting Antimicrobials in *Enterococcus faecium*. *Antimicrob Agents Chemother* **61** (2017).
9. D. Vesic, C. J. Kristich, A Rex family transcriptional repressor influences H2O2 accumulation by Enterococcus faecalis. *J Bacteriol* **195**, 1815-1824 (2013).
10. C. A. Mascari, J. L. Little, C. J. Kristich, PASTA-kinase-mediated signaling drives accumulation of the peptidoglycan synthesis protein MurAA to promote cephalosporin resistance in *Enterococcus faecalis*. *Mol Microbiol* 10.1111/mmi.15150 (2023).
11. H. Snyder, S. L. Kellogg, L. M. Skarda, J. L. Little, C. J. Kristich, Nutritional control of antibiotic resistance via an interface between the phosphotransferase system and a two-component signaling system. *Antimicrob Agents Chemother* **58**, 957-965 (2014).
12. D. Vesic, C. J. Kristich, MurAA is required for intrinsic cephalosporin resistance of *Enterococcus faecalis*. *Antimicrob Agents Chemother* **56**, 2443-2451 (2012).
13. N. E. Minton, D. Djorić, J. L. Little, C. J. Kristich, GpsB Promotes PASTA Kinase Signaling and Cephalosporin Resistance in *Enterococcus faecalis*. *J Bacteriol* **204**, e00304-00322 (2022).
14. C. L. Hall *et al.*, Structure and Dimerization of IreB, a Negative Regulator of Cephalosporin Resistance in *Enterococcus faecalis*. *J Mol Biol* **429**, 2324-2336 (2017).
15. D. Djorić, C. J. Kristich, Extracellular SalB Contributes to Intrinsic Cephalosporin Resistance and Cell Envelope Integrity in Enterococcus faecalis. *J Bacteriol* **199** (2017).
